# ApoNETosis: discovery of a novel form of neutrophil death with concomitant apoptosis and NETosis

**DOI:** 10.1038/s41419-018-0846-9

**Published:** 2018-08-06

**Authors:** Dhia Azzouz, Nades Palaniyar

**Affiliations:** 1 Program in Translational Medicine, Peter Gilgan Centre for Research and Learning, The Hospital for Sick Children, Toronto, ON Canada; 2 Department of Laboratory Medicine and Pathobiology, University of Toronto, Toronto, ON Canada; 3 Institute of Medical Sciences, Faculty of Medicine, University of Toronto, Toronto, ON Canada

## Neutrophils are short-lived cells that can undergo many types of cell death

**Fig. 1:**
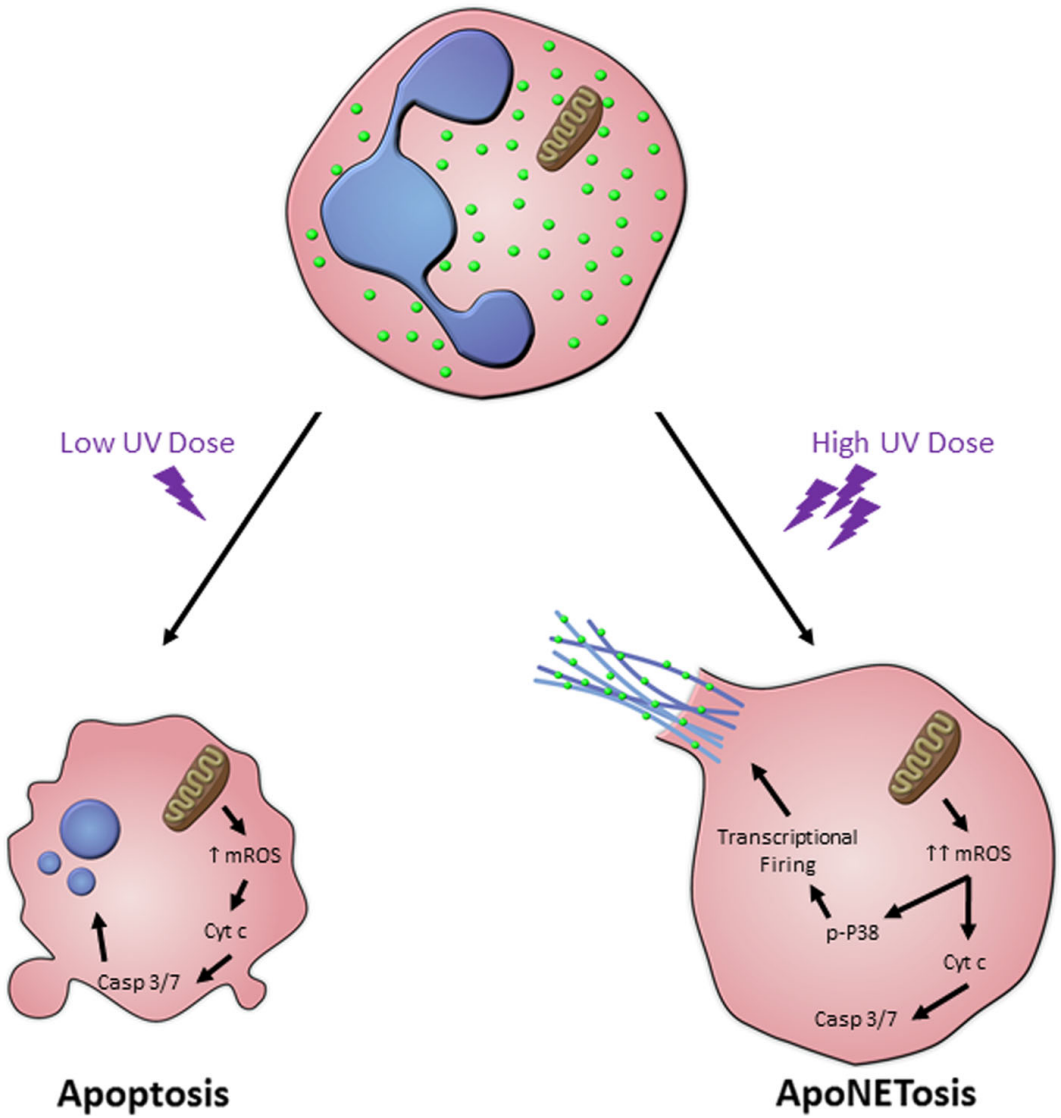
The key steps of UV-induced apoptosis and ApoNETosis. Low dose UV irradiation induces low levels of mitochondrial ROS, cytochrome c release, caspase cascade activation, and finally nuclear condensation and blebbing. On the other hand, high dose UV irradiation results in large amounts of mitochondrial ROS, cytochrome c release, and caspase cascade activation. However, p38 activation and transcriptional firing are also induced, resulting in MPO-coated NET formation and NET release.

Neutrophils are the most abundant leukocyte in the human body. They have a short lifespan (average of 7 hours to 5.4 days) and types of neutrophil death affect many inflammatory and autoimmune disease states. Neutrophils have classically been known to die by apoptosis. A novel form of neutrophil death leading to the release of extracellular traps (NETs) that is different from apoptosis was discovered in 1996^1^. In the past decade, mechanistic details of NET formation (NETosis) have been studied in greater detail^2,3^. NETosis has now been defined as a unique form of cell death that is completely independent from apoptosis^1,2^. We have recently reported that higher doses of ultraviolet (UV) induce both apoptosis and NETosis in the same neutrophil, in Cell Death Discovery^4^. In this novel form of neutrophil death, ApoNETosis, NETotic steps override certain key apoptotic steps (Fig. 1). ApoNETosis, apoptosis, NETosis, and other forms of neutrophil death have unique similarities and differences, and understanding molecular steps regulating ApoNETosis could help to explain certain pathobiological conditions.

## Apoptosis and necrosis are the classical forms of neutrophil death

There are two types of neutrophil apoptosis: intrinsic and extrinsic apoptosis. Intrinsic apoptosis is induced primarily by UV light and bacteria^5,6^. Following exposure to UV, mitochondrial membrane is destabilized resulting in the release of cytochrome c^7^. Once in the cytoplasm, cytochrome c binds the adapter protein Apaf1. The complex activates pro-caspase 9 to form caspase 9. Caspase 9 then activates the effector proteins such as caspase 3/7, which then activates caspase-activated DNase (CAD)^7^. Extrinsic apoptosis is induced primarily by Fas ligand and tumor necrosis factor α (TNFα)^8^. Fas and tumor necrosis factor receptor-1 (TNFR-1) are membrane proteins that are activated by Fas ligand and TNFα, respectively^8^. Activation of either membrane protein results in the formation of the death-inducing signaling complex (DISC) composed of Fas, Fas-associated protein with Death Domain (FADD), and pro-caspase 8. This results in the activation of caspase 8, which in turn activates effector caspases 3/7 followed by the activation of CAD^8^. Depending on phosphorylation state, caspase 8 has also been shown to prolong survival of neutrophils^6^. Bcl-2 family members play important roles in neutrophil apoptosis. Neutrophils express high levels of pro-apoptotic Bcl-2 family members, Bax and Bak, which may play a role in their observed short lifespans^9^. Pro-apoptotic Bid, Bim, and Bad are also expressed in neutrophils^9^. Anti-apoptotic Bcl-2 family members, Mcl-1, Bcl-xl, and A1, are expressed in neutrophils and may be increased by survival factors^9^.

There are many factors and signaling pathways that affect neutrophil apoptosis. granulocyte/macrophage colony-stimulating factor, granulocyte colony-stimulating factor, and ATP are a few of the factors that promote neutrophil survival^9^. On the other hand, activation of certain tumor necrosis factor/nerve growth factor receptor family members can result in enhanced apoptosis^9^.

While apoptosis is highly regulated, necrosis can be uncontrolled and often accidental. It is often the result of stresses such as osmotic shock, heat and freeze thawing. Higher levels of bacteria have also been reported to induce necrosis in neutrophils. There is a regulated type of necrosis termed necroptosis, which can be induced by bacterial pathogen-associated molecular patterns and viral infections. Necroptosis is dependent on RIPK1 and RIPK3 activation and negatively dependent on caspase 8^10^.

## NETosis is a cell death that is specific to neutrophils

A form of cell death specific to neutrophils is NETosis. NETosis, which is independent of apoptosis and necrosis, was first observed by Takei *et al* in 1996^1^. The research team induced NETosis by exposing the neutrophils to PMA^1^. The bactericidal activity of NETs was uncovered by Brinkmann *et al*^2^. Since its discovery, two types of NETosis have been characterized^3,11^. The first type is NADPH oxidase 2 (NOX2)-dependent NETosis, which is induced by PMA, LPS, and various types of bacteria such as *Pseudomonas aeruginosa*. During NOX-dependent NETosis, ROS is produced by NOX2, mitogen-activated protein kinases (MAPKs: ERK, p38, and JNK) are activated, transcriptional firing is initiated and ultimately NETs are released^11–13^. The second type is NOX-independent NETosis, which is induced by calcium ionophores (A2317 and ionomycin) some crystals, certain microbes, and UV light^4,11^. During NOX-independent NETosis, increased ROS is produced by the mitochondria, MAPK p38 is activated, transcriptional firing is increased and ultimately NETs are released^3,13^. Specific to NETosis induced by calcium ionophores, intracellular calcium increase results in activation of peptidylarginine deiminase 4 (PAD4), which citrullinates histones at promoter regions that plays a role in accelerating the formation of NETs^3,14^. While NETosis has been classically seen as a type of cell death, a non-lytic form of NETosis has been uncovered and termed as vital NETosis^15^.

## ApoNETosis: simultaneous apoptosis and NETosis in the same neutrophil

The classical thinking has been that NETosis and apoptosis are two distinct processes. In regards to the interaction between apoptosis and NETosis, caspase activity (a hallmark of apoptosis) was reported to be lacking during induction of NETosis^1,2^. No incidence of simultaneous induction of apoptosis and NETosis in the same neutrophil has been previously reported. This highlights the significance of the uncovering of the ability of UV, at higher doses, to induce NETosis and apoptosis simultaneously in the same neutrophil in a novel form of cell death (Fig. 1). ApoNETosis, similar to apoptosis and all types of NETosis, requires increased ROS production, specifically mitochondrial in origin in the case of ApoNETosis. NOX is not active during UV-induced ApoNETosis, indicating that it is NOX-independent. Activation of MAPK p38 is required for UV-induced ApoNETosis. Similar to other forms of NOX-independent NETosis, ApoNETosis requires transcriptional firing to take place^4^. However, it differs from other forms of NOX-independent NETosis in that histones are not citrullinated. This suggests that there is no increase in intracellular calcium concentration and PAD4 activation during UV exposure of neutrophils. Similar to other types of apoptosis, caspase 3 is cleaved during UV-induced ApoNETosis, indicating that apoptotic pathways are activated. However, nuclear blebbing does not occur during ApoNETosis. Instead DNA is released in NET structures as seen during NETosis.

## Significance and future directions

NETosis is a form of cell death that is independent of apoptosis and necrosis. We uncovered that UV at higher doses can induce both apoptosis and NETosis in the same neutrophils in a novel process termed ApoNETosis. High dose UV as a tool for generating NETs for NET clearance studies is promising since drawbacks of using chemicals, toxins, or cytokines are not present in this experimental approach. The discovery that UV can induce ApoNETosis may shed light on states of inflammation observed following exposure to UV. Further mechanistic details and pathological relevance of ApoNETosis remain to be established.
